# Variation of *Burkholderia cenocepacia* cell wall morphology and mechanical properties during cystic fibrosis lung infection, assessed by atomic force microscopy

**DOI:** 10.1038/s41598-019-52604-9

**Published:** 2019-11-06

**Authors:** A. Amir Hassan, Miguel V. Vitorino, Tiago Robalo, Mário S. Rodrigues, Isabel Sá-Correia

**Affiliations:** 1 iBB – Institute for Bioengineering and Biosciences, Instituto Superior Técnico, Universidade de Lisboa, Lisbon, 1049-001 Portugal; 2 Department of Bioengineering, Instituto Superior Técnico, Universidade de Lisboa, Lisbon, 1049-001 Portugal; 3 BioISI – Biosystems and Integrative Sciences Institute, Faculdade de Ciências, Universidade de Lisboa, 1749-016 Lisboa, Portugal; 4 Departamento de Física, Faculdade de Ciências, Universidade de Lisboa, 1749-016 Lisboa, Portugal

**Keywords:** Nanoscale biophysics, Nanoscale biophysics, Bacteriology, Bacteriology, Clinical microbiology

## Abstract

The influence that *Burkholderia cenocepacia* adaptive evolution during long-term infection in cystic fibrosis (CF) patients has on cell wall morphology and mechanical properties is poorly understood despite their crucial role in cell physiology, persistent infection and pathogenesis. Cell wall morphology and physical properties of three *B*. *cenocepacia* isolates collected from a CF patient over a period of 3.5 years were compared using atomic force microscopy (AFM). These serial clonal variants include the first isolate retrieved from the patient and two late isolates obtained after three years of infection and before the patient’s death with cepacia syndrome. A consistent and progressive decrease of cell height and a cell shape evolution during infection, from the typical rods to morphology closer to cocci, were observed. The images of cells grown in biofilms showed an identical cell size reduction pattern. Additionally, the apparent elasticity modulus significantly decreases from the early isolate to the last clonal variant retrieved from the patient but the intermediary highly antibiotic resistant clonal isolate showed the highest elasticity values. Concerning the adhesion of bacteria surface to the AFM tip, the first isolate was found to adhere better than the late isolates whose lipopolysaccharide (LPS) structure loss the O-antigen (OAg) during CF infection. The OAg is known to influence Gram-negative bacteria adhesion and be an important factor in *B*. *cenocepacia* adaptation to chronic infection. Results reinforce the concept of the occurrence of phenotypic heterogeneity and adaptive evolution, also at the level of cell size, form, envelope topography and physical properties during long-term infection.

## Introduction

The Gram-negative opportunistic bacterial pathogens *Pseudomonas aeruginosa* and *Burkholderia cepacia* complex (Bcc) exhibit extensive genetic and phenotypic heterogeneity during persistent infection and evolution in the lungs of cystic fibrosis (CF) patients over the years^1–4^. The molecular mechanisms underlying adaptation to the lung and genotypic and phenotypic diversification have been intensively studied in the more prevalent CF pathogen *P*. *aeruginosa*^3,4^. However, Bcc lung infections in CF are highly feared because they are associated with poor prognosis and increased risk of death due to rapid lung function deterioration and, in certain cases to a necrotizing pneumonia, bacteraemia, and sepsis, (the cepacia syndrome)^5–8^.

During long-term lung infection in CF patients, *P*. *aeruginosa* and Bcc bacteria face multiple selective pressures in the highly challenging, fluctuating, and stressful environment of the patients´ airways, in particular due to antimicrobial therapy, the action of the host immune system and of other members of the microbiome and the decrease of oxygen availability as the result of lung function deterioration^9,10^. Under those stresses, several genetic changes accumulate in the initial infecting bacterial strain leading to phenotype and genotype heterogeneity. CF bacterial pathogens phenotypic diversification can be recognized in terms of colony morphology diversity^11–17^ and variation of clinically relevant phenotypes such as antibiotic resistance^11,17–20^, ability to form biofilms^16,21–24^, virulence potential^14,25–27^, among many others^12,17,28–32^. Remarkably, such phenotypic heterogeneity within human hosts has important clinical implications. For example, antimicrobial susceptibility diversity within the bacterial population isolated from an individual sputum sample may affect the treatment of life-threatening infections given that the results from antimicrobial testing carried out on single isolates randomly collected can be a poor predictor of the clinical outcome of antibiotic therapy^7,18,19^.

Bacterial cell envelope plays a central role in cell physiology and the alteration of surface properties can implicate the variation of phenotypes that play a crucial role in the pathogenesis of infectious diseases, such as antibiotic resistance and biofilm formation^28,32,33^. However, very few bacterial species have been on the focus of studies related to cell surface physical properties^33–35^ and information on the diversification and adaptive evolution at the level of Bcc bacteria cell wall mechanical properties during CF chronic lung infections is missing. In this context, over the last years atomic force microscopy (AFM) emerged as an essential tool for understanding the nanomechanics of live systems^36–38^. Hence, the objective of the present study was to obtain this knowledge by studying cell surface morphology and mapping the mechanical properties of *Burkholderia cenocepacia* clonal variants isolated from the lungs of a CF patient during long term infection using AFM. The *B*. *cenocepacia* isolates examined are from a collection of 11 serial clonal variants obtained from the same CF patient over a period of 3.5 years, from the onset of infection until the patient’s death^11,39^. The clonal variants tested were: IST439, the first isolate retrieved; IST4113, obtained three years later after an exacerbation with the patient hospitalization and treatment with intravenous therapy with gentamicin and ceftazidime and found to be highly resistant to different classes of antimicrobials; and IST4134, obtained 3 months later, just before the patient’s death with cepacia syndrome^11,39,40^. These isolates were picked at random from selective agar plates obtained in the major Portuguese CF Center at Hospital de Santa Maria during consultation routines. The clinical isolates examined are of high interest in the context of this study because they were previously characterized by phenotypic^11^, transcriptomic^40^, proteomic^27,41^ and metabolic profiling^42^. Results on the comparison of the virulence potential of these isolates using non-mammalian infection models and of their ability to modulate dendritic cell function are also available^25,43^. The two late variants were found to have lost the ability to produce the OAg molecule of the lipopolysaccharide^44^ present in the early isolates and to be more internalized by dendritic cells and show improved survival within dendritic cells when compared to the initial isolate^43^. Inflammatory cytokines were highly expressed in all the sequential clonal isolates but this pro-inflammatory trait was more pronounced in dendritic cells infected with the late variants compared with the isolate retrieved at the first stages of infection^43^.

Results of the present study, in which AFM cell wall morphology and mechanical properties of these three sequential *B*. *cenocepacia* clonal variants were studied, reinforce the concept of the occurrence of phenotypic variation and adaptive evolution also at the level of cell size, form, envelope topography and physical properties during long-term infection.

## Results

### *B*. *cenocepacia* morphology and surface roughness evolution during long-term CF lung infection

**Figure 1:**
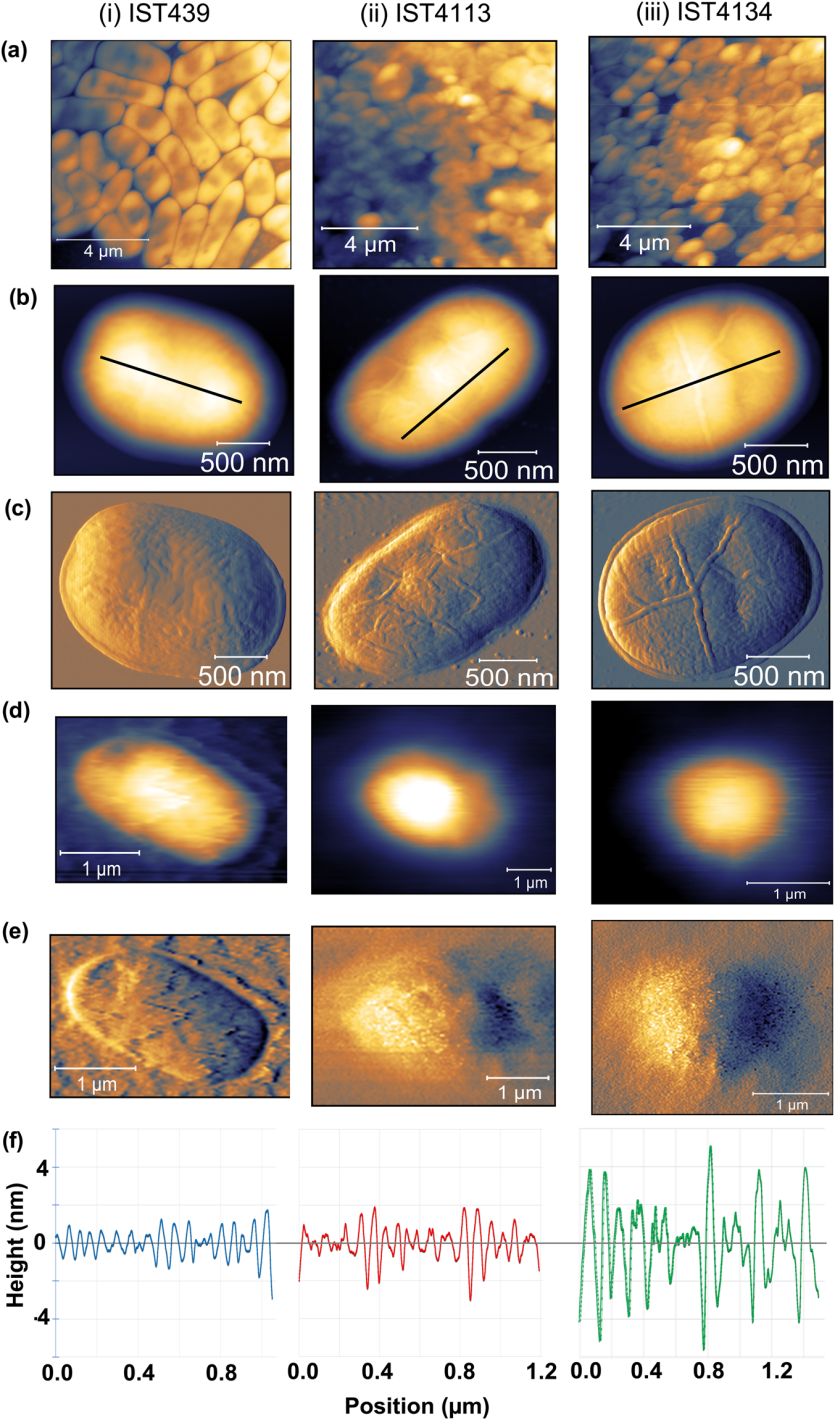
Cell topography and surface roughness. AFM images of the studied clonal variants (i - IST439, ii - IST4113 and iii - IST4134) show (**a**) topography of biofilm; (**b**) topography of planktonic and (**c**) corresponding deflection images in air; (**d**) topography of planktonic and (**e**) corresponding deflection in liquid samples; (**f**) representative roughness profiles (marked by the black lines) for the same variants. The late variants exhibit distinct string-like surface features, whereas both the images and roughness profile of the IST439 variant show a smoother surface.

The cell morphology and topography of the three *B*. *cenocepacia* clonal variants were examined using AFM in both planktonic and biofilm forms. The images for individual cells were obtained in air and in liquid environment. Concerning cell topography in air (Fig. 1), the easiest way to clearly visualize individual cells, the three clonal variants examined in their planktonic form exhibit the porous network architecture of the cell wall previously reported by others^45,46^. However, late variants IST4113 and IST4134 show features, characterized by string-like formations on the surface of the bacteria, not found in the early isolate IST439. Specifically, the last isolate retrieved from the patient, IST4134, displays longer and well-organized string-like structures that span the entire length of the cell while IST4113 exhibits shorter and less organized structures (Fig. 1(b,c)). However, the same structures could not be observed in the biofilm images Fig. 1(a). The surface roughness of the cells examined in air in their planktonic form was assessed by defining longitudinal cross sections along the cell surface, as shown in Fig. 1(b,f). The first isolate, IST439, was found to be smoother, with average roughness (root mean square of the cross sections) of 0.9 ± 0.1 nm. The late variants showed an average roughness of 1.0 ± 0.1 nm and 2.0 ± 0.1 nm respectively. Alternatively, we have also measured the roughness by taking the root mean square over an area defined on the top of the cell after subtracting the cell envelope. The resulting values for the first, second and third isolate were respectively 1.7 nm, 2.0 nm and 2.5 nm with 0.5 nm standard deviation. We find the first method less prone to errors because it is easier to separate the cell roughness from the cell contour/envelope - nonetheless, both methodologies indicate the same trend. The surface roughness from the images obtained in liquid was impossible to visualize due to the poor resolution of the images seemingly caused by some mobility of either the cell or the cell surface. We have used both contact and tapping mode but the results were similar.

**Figure 2:**
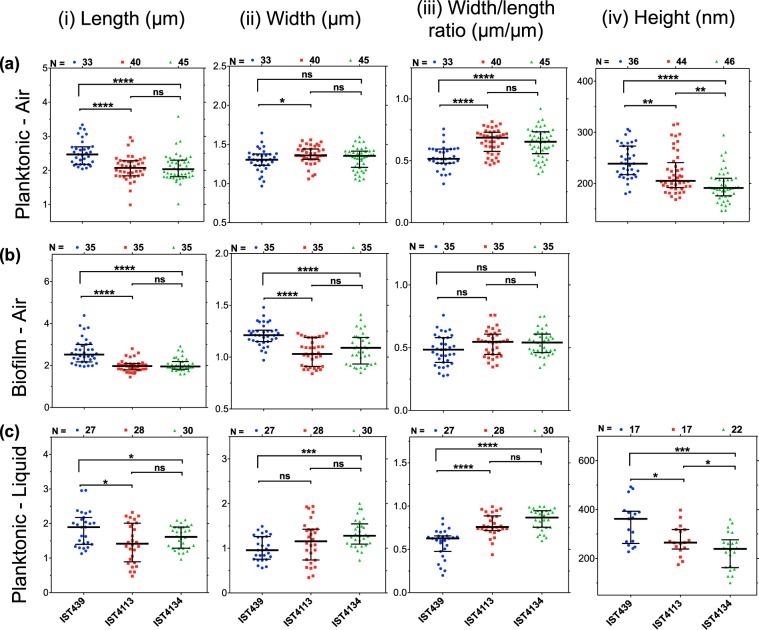
Cell morphology. Distribution of the cell dimensions (i - length, ii - width, iii - width/length ratio and iv - height) measured with AFM for N individual cells of the studied clonal variants (blue circle - IST439, red square - IST4113 and green triangle - IST4134), for (**a**) planktonic and (**b**) biofilm growth conditions both measured in air and for the (**c**) planktonic form measured in liquid environment. An increase of the width/length ratio and a decrease of cell height, as well as the evolution of the cell shape during long-term infection from a rod-like to a more cocci-like morphology can be observed. The results of the Mann-Whitney u-test (^*^P < 0.05, ^**^P ≤ 0.005, ^****^P ≤ 0.0005, ns not significant) are indicated.

Concerning cell dimensions measurements, the late variants, IST4113 and IST4134, were found to be significantly smaller in length than the early isolate IST439, but no significant differences were found between the lengths of late variants, as shown on Fig. 2(i). Similar significant differences of the cell length were observed both in air and liquid, however the absolute values registered in liquid environment were smaller than those in air (Fig. 2(i)). Additionally, either in air or in liquid environment, the height of the cells also decreases from the first to the last clonal isolate by about 30% (Fig. 2(iv)). Overall, the observed trend points towards an evolution of the cell shape from rod to a more rounded shape, with the ratio width/length increasing from around 0.52/0.62 (air/liquid) for the early isolate to about 0.68/0.75 (air/liquid) and 0.65/0.86 (air/liquid) for the second and third isolates, respectively (Fig. 2(iii)). The AFM images of the biofilm (Fig. 1(a)) show cell dimensions consistent with the reduction of the cell size and increase of the ratio width/length (Fig. 2(iii, panel b)) observed for the individual cells. However, due to the fact that cells are tightly packed it is more difficult to accurately determine cell dimensions in biofilm.

### Surface and mechanical properties evolution during long-term CF lung infection

**Figure 3:**
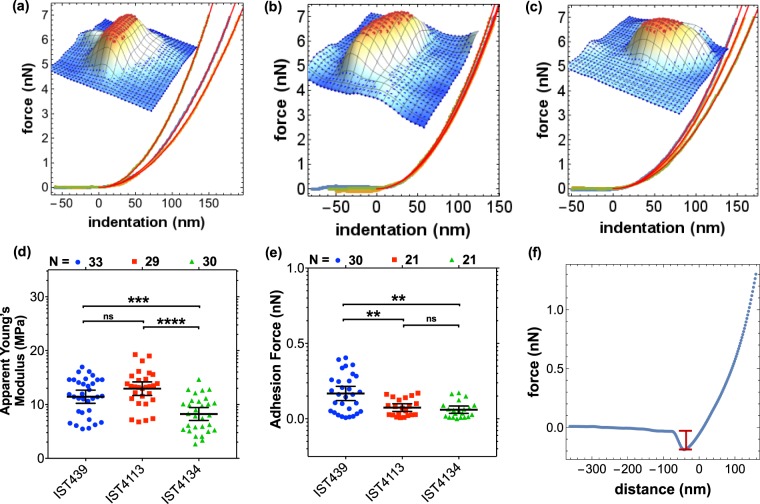
Elasticity and adhesion studied by AFM in liquid samples. (**a**–**c**) Example of indentation curves and respective fits using Sneddon model for the first, second and third clonal variants with insets showing the respective 3D maps and the selection of points at the top of the cell; (**d**,**e**) distribution of young modulus and adhesion forces respectively, for each isolate; (**f**) a retract curve obtained for the first isolate illustrating the adhesion measurement. The results of the Mann-Whitney u-test (^*^P < 0.05, ^***^P ≤ 0.001, ^****^P ≤ 0.0005, ns not significant) are indicated.

Cell elasticity was examined at selected points (inset of Fig. 3(a–c)) along the cell surface and the resulting force-distance curves were compared with the Sneddon contact mechanics model (Fig. 3(a–c)), as described in the Methods section. All these measurements were performed in liquid environment because the measurement of the mechanical properties of dried cells is questionable^47–51^. In fact, when measurements were made in air, the apparent Young’s modulus of the cell surface was about 40 times larger.

Concerning the elasticity, and in particular the apparent Young’s modulus of the cell surface measured in liquid environment, the values show a significant decrease from the early isolate to the late variant (Fig. 3(d)), but the highly antibiotic resistant clonal variant IST4113^11^ exhibited the highest values. Concerning the adhesion of the bacteria surface to the Si_3_N_4_ AFM tip (Fig. 3(e)), the first isolate was found to adhere better than the late isolates whose lipopolysaccharide (LPS) structure loss, during CF infection, the O-antigen (OAg) present in the early isolate^44^. No significant differences were found between the adhesion of the two late isolates missing the OAg, Fig. 3(e).

### Growth curves of the *B*. *cenocepacia* clonal variants under aerobic or microaerophilic conditions

**Figure 4:**
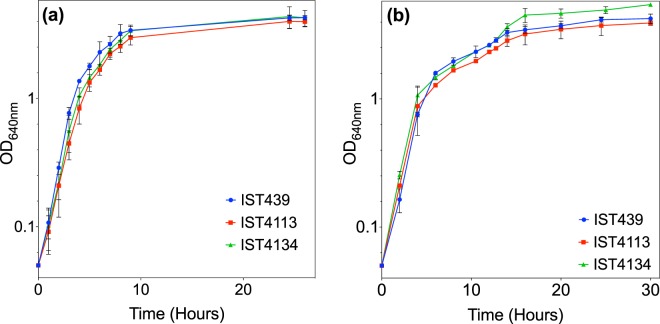
Growth under oxygen limitation. Evolution of the optical density values during the incubation time in the same medium (LB) for the three clonal variants examined under (**a**) aerobic condition and (**b**), microaerophilic conditions. Experimental values and error bars represent the mean and the estimated standard deviation, respectively, for three independent growth experiments. These results indicate that the late clonal variant grows better under microaerophilic conditions whereas the early IST439 grows better under aerobic conditions.

The growth curves of the three clonal variants examined were compared in the same Lysogeny broth (LB) medium under aerobic and microaerophilic conditions, at 37 °C (Fig. 4). The general conclusion is that under aerobic conditions, the growth performance of the first isolate is slightly better (higher specific growth rate and higher final biomass concentration attained) than the late isolates, with the highly antibiotic resistant intermediary isolate (IST4113) exhibiting the slowest and less efficient growth. This behaviour contrasts with the growth performance observed under microaerophilic conditions which are conditions closer to those expected to occur in the CF patient lung, especially at late stages of disease progression and very low values of Forced expiratory volume in one second (FEV1)^52^. Although the reported differences are small, the early isolate consistently exhibited, under oxygen limitation, the lowest specific growth rate while the last isolate showed the more rapid growth and efficient biomass production suggesting that the late isolates are better adapted to the CF lung. Moreover, under microaerophilic conditions, the growth curves of the 3 isolates exhibit a pattern consistent with diauxic growth in the complex LB medium, a behaviour observed before (unpublished data).

### Biofilm growth of *B*. *cenocepacia* clonal variants

**Figure 5:**
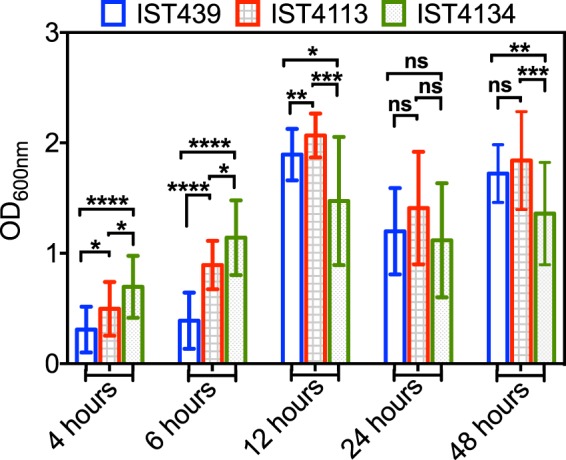
Biofilm formation. Quantification of the biofilm formed at different incubation times, based on the OD_600nm_ values of crystal violet-stained biomass. Bar height and error bars represent the mean and standard deviation obtained from three independent growth experiments with 7 measurements each. The Mann-Whitney u-test (^*^P < 0.05, ^***^P ≤ 0.001, ^****^P ≤ 0.0005, ns not significant) values are indicated.

The quantification of biofilms in terms of biomass using crystal violet staining after 4 and 6 hours of incubation is consistent with the specific growth rates and final biomass attained by the three clonal variants, grown under microaerophilic conditions in the planktonic lifestyle (Figs. 4 and 5). In fact, the late isolates produce more rapidly immature biofilms of higher biomass, formed after 4–6 hours. However, the relative biomass of the mature biofilms formed after 24–48 h of incubation is consistent with the level of exopolysaccharide produced by the three variants, as reported before for the same isolates/growth medium^11^, being maximal for the intermediary isolate.

## Discussion

During long term infection, the genetic adaptation of bacteria of the *Burkholderia cepacia* complex (Bcc) to the challenges of multiple selective pressures occurring in the cystic fibrosis (CF) airways is known to take place^1,2,53–55^. The present study provides the first insights into the adaptive evolution of these bacteria at the level of cell size, form, envelope topography and physical properties during long-term infection. However, it cannot be guaranteed that the properties reported here are identical to those exhibited by bacteria grown in the lungs and measured in their native environment. Given the highly relevant information gathered over the years on the three sequential *B*. *cenocepacia* variants examined here, it is possible to speculate on how the variation in surface properties can impact phenotypes of clinical relevance in the pathogenesis of infectious diseases, such as adhesion, resistance to antibiotics, biofilm formation and growth efficiency in the CF environment.

Former studies have shown that in *B*. *cenocepacia* the O-antigen (OAg) of the lipopolysaccharide (LPS), occurring in the outermost layer of the cell, is lost or modified during persistent infection of the lungs^32,44^. The loss or modification of the OAg appears to play an important role during the infection process, in particular in the colonization step (adherence) and ability to overcome host defence mechanisms^32,56,57^. Recently, we have shown that the tendency of the most prevalent and feared species *B*. *cenocepacia* and *B. multivorans* to lose the OAg during chronic infection is higher than the one of the rarely found *B*. *cepacia* and *B*. *contaminants* that keep the OAg even during decades of infection^58^. Moreover, *B*. *cenocepacia recA* lineage IIIA strains, as it is the case of the isolates examined in the present study known to lead to particularly destructive infections, exhibit the most frequent OAg loss, compared with lineage IIIB^58^. Concerning the clonal isolates tested in the present study, the OAg is only present in the early isolate IST439^44^. Mutations within the OAg cluster of the serial isolates examined were identified but none of them could definitely be associated to OAg loss^44^. In the present study, we found that the ability of the earlier isolate to adhere to the AFM Si_3_N_4_ tip, when assessed in liquid environment, was significantly higher than the estimated ability of the late variants, lacking the OAg whose adhesion values were similar. These results are consistent with the concept that the variability in the LPS OAg affects bacterial adherence and colonisation and the ability to evade the host’s defence mechanisms being an important factor in *B*. *cenocepacia* adaptation to chronic infection^32,44,58–60^.

In agreement with the characterization of Gram-negative sacculus^61,62^, images in air have clearly revealed tube-like features not regularly spaced mostly in the plane of the sacculus and roughly perpendicular to the long axis of the cell, for the two late isolates, but not for the early isolate. These formations are short and organized randomly in IST4113 cell surface but well developed in IST4134. These features do not seem to be associated to the division septum and its equatorial rings, as previously reported in studies in which the sacculus of *Escherichia coli* and *Bacillus subtilis*^63,64^, of *Streptococci* and *Enterococci*^65,66^ and *Staphylococcus aureus*^67^ was analysed. With very few exceptions, the chemical and biological bacterial components studied that contribute to cell mechanics are related with the peptidoglycan layer of the cell envelope and changes in its structure^33,34,68^. Apparently, the peptidoglycan is made up of circumferential oriented bands of material interspersed with a more porous network^45^. Peptidoglycan is the largest component of the bacterial cell wall determining the shape and preserving its integrity^62^. Its elastic nature helps withstand stretching forces caused by bacterial turgor pressure. The reported bands were proposed to define regions with different availability for insertion of new peptidoglycan^45,46^. Interestingly, indentation tests performed in liquid show that the surface elasticity modulus, decreases significantly from the early isolate to the last clonal variant. However, the highly antibiotic resistant intermediary isolate IST4113^11^ expressed maximal values. Higher rigidity and increased elasticity was recently reported to be associated with a lower outer membrane permeability which may lead to the reduction of antibiotic diffusion into the cells^69^.

The suggested remodelling of cell surface of the three clonal variants examined in this study during long term infection was already anticipated based on the results of the comparison of genomic expression of these same clonal variants using transcriptomic and quantitative proteomic analyses^27,40,41^. These studies have shown differences in the level of expression of genes/proteins involved in the biogenesis of cell envelope and outer membrane in the three variants, among the several hundred of genes found to be differentially transcribed in the late isolates compared to the early isolate. These genome-wide expression results reflect a marked reprogramming of genomic expression at different levels^27,40,41^, including the alteration of bacterial cell surface that contributes to the intrinsic and acquired resistance of Bcc bacteria to antibiotics. Remarkably, it was found that the late isolates are significantly more resistant to a wide range of antibiotics, with isolate IST4113 displaying the higher resistance levels^7,11,27,40^. Recent genomic studies strengthened the concept that cell wall remodelling relates with the alteration of bacterial mechanical properties^33,35^. For example, *E*. *coli* mutants deleted for genes encoding proteins associated with cell-wall synthesis exhibit different stiffness defects^35^ and the accumulation of the peptidoglycan *D*-Alanine residues is tightly regulated in *P*. *aeruginosa* since their accumulation reduces peptidoglycan cross-linking and cell stiffness^33^.

In this study, we clearly observed a consistent and progressive pattern of decrease of the height and the increase of the width/length ratio of *B*. *cenocepacia* cells during long term infection, both in air and in liquid environments. Although the *B*. *cenocepacia* clonal isolates examined may not be representative of the expected population heterogeneity present at each isolation time in the CF lung, the consistency of the pattern strongly suggests that *B*. *cenocepacia* underwent convergent evolution towards the minimization of bacterial size during infection. Moreover, besides the decrease of the size of *B*. *cenocepacia* cells during infection, the bacterium underwent a cell shape evolution from the typical rod form of the species to a cell morphology closer to the spherical form of cocci. The referred pattern was observed for both the planktonic and biofilm growth mode. This same pattern was described before for two nasopharyngeal bacterial pathogens during adaptation to human mucosa and the authors hypothesized that this transition was selected to reduce the cell surface sensitivity to immune attacks given that the ratio surface/volume is smaller than that of bacilli^70^. Consistent with this hypothesis, several studies have shown the relevance of cell surface size when bacterial cells are facing immune attacks, small microbial size allowing a more efficient evasion of host defences^71–73^. Microbial cell size appears to be an important pathogenesis factor and minimization of bacterial size was demonstrated to be a mechanism used for example by *Streptococcus pneumoniae* to circumvent complement-mediated killing by the host^73^. The cell shape modification from rods to cocci-like form has been very-recently reported to occur in response to antibiotic stress in multi-drug resistant *E*. *coli*^74^ and has been genetically and biochemically demonstrated to occur during the prolonged antibiotic selective pressure that is extensively and aggressively administered to CF patients chronically infected with *P*. *aeruginosa* bacteria^71^. Remarkably, in the particular case of the CF patient from whom the examined isolates were obtained, the clinical situation was significantly deteriorated being hospitalized and submitted to intensive intravenous antibiotic therapy immediately before IST4113 isolation^11,39^.

Many Gram-negative pathogens alter their characteristic rod-shaped forms to smaller coccoid-like forms after incubation for days to weeks in fresh or salt water and in nutrient poor environments bacteria tend to be much smaller in size than those grown in laboratory cultures^71^. Free-living cells tend to be smaller in nutrient poor environments because the acquisition of nutrients relies on diffusion and capture of molecules at the surface of the cell. In *E*. *coli*, cell size was found to be reduced by a factor of 3 in response to nutrient starvation^75^, *E*. *coli* adjusting size and growing larger and faster in nutrient-rich media compared with nutrient-poor media^75–78^. During the continuous and rapid deterioration of lung function, as the disease progresses, the oxygen concentration levels in the CF airways suffer a marked decrease^3,26^. Responses of *P*. *aeruginosa* to oxygen limitation indicate that this species growth in the CF lung is by aerobic respiration^79^ and the same metabolism was proposed for *B*. *cenocepacia*^11,40,80^. According to the hospital records, when the early isolate IST439 was obtained, the FEV1 value (the forced expiratory value in the first second) was 22% but no further values of FEV1 are available due to the subsequent severe deterioration of pulmonary function^11,52^. The fact that the late variants appear to grow more efficiently under microaerophilic conditions, while the early isolate exhibits the most efficient growth when in aerobiosis, supports the hypothesis of an adaptation of the late variants to severe oxygen depletion. The more adapted growth of the late isolates to oxygen-limitation is also consistent with the biomass increase of the biofilms resulting from growth during the first hours (4 and 6 hours) following initial bacteria adhesion. After 12–48 hours of growth, with the maturation of the biofilms formed, other mechanisms take over as it is the case of exopolysaccharide (EPS) production capacity^11,23,24^, the biomass concentration of the biofilms formed correlating well with the levels of EPS produced by each clonal variant^11^. Differences observed between IST4113 and IST4134 growth curves under both microaerophilic and aerobic conditions are likely the result of IST113 resistance to multiple antibiotics, resistance to which a fitness cost is associated^81,82^.

In summary, independently of the selective pressures that drive *B*. *cenocepacia* cell size and shape alterations during chronic infection of the lungs, it is likely that the adaptive evolution registered in this study may lead to a better performance under limiting oxygen concentration, to more efficient nutrient acquisition and to evasion of the host complement deposition, favouring persistent infection and pathogenesis. The positive correlation observed between cell shape change and elasticity modulus indicates that elasticity of the cell wall may play a key role in this adaptation process. Results from former genome wide expression analyses and extensive phenotyping of the isolates here examined have provided clues that strongly suggest a genetic adaptation to the challenges exerted by the immune system, antimicrobial therapy and nutrient and oxygen availability^27,40,41^. The shape and size evolution observed in this study is considered part of such metabolic reprogramming that leads to *B*. *cenocepacia* persistence in the CF lung^27,40,41^. Understanding the underlying adaptation mechanisms is essential also for an improved therapeutic outcome of long term infections in CF patients.

## Methods

### Bacterial strains and growth conditions

The three *Burkholderia cenocepacia* clonal variants examined in this study (IST439, IST4113 and IST4134) were recovered, as part of the hospital routine, from the sputum of a CF patient under surveillance at the major Portuguese CF Center in the Hospital de Santa Maria, Centro Hospitalar Lisboa Norte (CHLN) EPE, from 1999 to 2002^11,25,39^. Studies involving these isolates were approved by CHLN ´ ethics committee and the anonymity of the patient was preserved. Informed consent was also obtained from all participants and/or their legal guardians. All the methods were performed in accordance with the relevant guidelines and regulations. Bacterial cultures are stored at −80 °C in 1:1 (v/v) glycerol. Bacterial growth was carried out in Lysogeny Broth, Lennox (LB; Conda, Pronadisa), at 37 °C and 250 rpm, or in LB agar plates obtained by supplementation of LB with 2% agar (Iberagar, Portugal). LB medium at 37 °C was also used in biofilm experiments.

### Preparation of the AFM samples

Bacterial cells used for AFM analysis were deposited onto the gelatin coated mica for the observations and measurements done in liquid environment and onto freshly cleaved mica surfaces for the observations done in air. *B*. *cenocepacia* isolates were cultured overnight in LB medium, at 37 °C with shaking at 250 rpm, and then sub-cultured until mid-exponential phase. Bacterial planktonic cells in suspension were collected by centrifugation and washed three times with phosphate buffer saline (PBS). For observations and measurements in liquid environment, the gelatin-coated mica was prepared and the bacterial immobilization was done as described before^69,83,84^. Two types of gelatin with different concentrations (0.25%, 0.5% and 1% (w/v)), porcine gelatin Sigma G-6144 and G-2625 and bovine gelatin Sigma G-9382, were tested^83^. Briefly, a gelatin solution was prepared by dissolving 0.25 g, 0.5 g or 1 g gelatin in 100 ml of deionized water at 90 °C and cooled to 60–70 °C prior to vertically dipping several discs of the freshly cleaved mica into the solution. Following optimization, gelatin G-6144 was found to allow the best immobilization effectiveness and used thereafter. The gelatin-G-6144-coated mica surfaces were supported on edge on a paper towel and then air dried overnight. 20–40 μl of the bacterial suspension in PBS (10^8^ CFU/ml) was applied onto a gelatin-coated mica surface after being sonicated in ultrasonic bath (40 kHz, 19 W – Branson, Model 200, NL) for ~5 min^84^. The sample was allowed to rest for 10–20 min before it was rinsed in PBS and imaged in the liquid cell of the AFM.

For the images/observations taken in air, the deposition of bacteria on the mica surfaces was carried out as described before^85,86^ and used for other Gram-negative bacteria^45,48,87^, with few modifications. In brief and during the optimization of the protocol, PBS and deionized water were tested to prepare the bacterial suspension aliquots prior to deposition on mica. Given that the samples prepared with PBS formed aggregates at the freshly cleaved-mica surface, whereas those prepared with deionized water did not, 10 μl of those aliquots in ddH_2_O, for observation done in air, with a final bacterial concentration of 10^8^ CFU/ml, were immobilized onto freshly cleaved mica surfaces and left to rest for 15–20 min. The mica surfaces were rinsed twice with deionized water to detach the weakly adherent and the non-adherent cells and allowed to dry before AFM analysis for another 15–20 min. The biofilm samples were prepared for AFM observation as described before^88^ with few modifications. The microtiter plates were incubated without shaking at 37 °C for 12 hours. The unattached planktonic bacteria were washed twice with sterile saline solution by pipetting in and out. The remaining biofilms were fixed by 10% formalin in PBS for 10 min. Next, the plates were inverted to remove all the fixation solution and the fixed biofilms were washed twice with sterile saline solution by pipetting in and out. Finally, the biofilm was resuspended in ddH_2_O, and 10 µl of the suspended biofilm was deposited into the freshly cleaved micas and air-dried for AFM imaging.

### AFM observations and measurements

Cell samples were analysed using a PicoSPM LE system of Molecular Imaging in a liquid cell containing PBS and in air at room temperature. Bruker MLCT-F microlevers with nominal cantilever stiffness of 0.6 N/m and nominal tip radius of 20 nm were used for all experiments. Images obtained in air were taken in contact mode whereas in liquid environment both contact and tapping modes were used depending on which proved best. To measure cell surface roughness 2.5 × 2.5 μm^2^ (approximately) images with 512 × 512 pixels were obtained. Twelve representative bacteria of each isolate were selected for roughness measurements.

For nanomechanics and adhesion measurements in liquid environment force spectroscopy maps, consisting of 32 × 32 approach/retract force-distance (FD) curves, were obtained over an area slightly larger than the cell footprint. The maximum cantilever deflection was set constant in all experiments, yielding a maximum applied force of roughly 15 nN. The tip-sample approach speed was also set constant, to 0.4 μm/s. To reduce bias due to different cantilevers being used on different populations, each cantilever was used to measure 2–3 bacteria of each isolate population and the order in which the different isolates was measured was randomized^89,90^. In total, about 40 bacteria of each population were measured in liquid environment and 14 cantilevers were used. Nanomechanical analysis was performed using a custom-made software. For each grid, we selected only curves obtained at the top of the bacteria (above 85% of the total height of the bacteria), as shown in the inset of Fig. 3(a–c). The contact part of the approach curves was analysed according to Sneddon contact model^36^ that establishes a relationship between load and indentation and from which it is possible to extract the reduced Young’s modulus Fig. 3(d). We have estimated the adhesion force Fig. 3(e) from the minimum of the retract part of the curves as illustrated in Fig. 3(f). For each cell, all curves obtained at the top of the cell were analyzed and the median was kept, then for each isolate sample we rejected values further away from 3 standard deviations. To determine the apparent Young’s modulus of the cell we assumed a non-deformable tip and a Poisson’s ratio of 0.5^36,91^. We assumed nominal values for the tip radius and used Sader method to calibrate the cantilever spring constant^92^.

### Growth curves

The growth curves of the three clonal variants examined under aerobic and microaerophilic conditions were monitored by measuring culture optical density at 640 nm (OD_640_). Cells were grown in LB medium at 37 °C in shaking flasks (100 ml with 30 ml of liquid medium) in an orbital shaker at 250 rpm (for aerobic growth) or standing in a microaerophilic atmosphere, containing 5–8% oxygen and 12–15% carbon dioxide, generated in sealed jars using the GENbox microaerator (bioMérieux, Marcy L’Etoile, France). Results are from three independent growth experiments.

### Biofilm formation assays

Biofilm formation assays were based on a described methodology^11,93^. Overnight liquid cultures of each CF isolate were transferred to LB medium and grown at 30 °C with orbital agitation until the mid-exponential phase was reached. The cultures were subsequently diluted to a standardized culture OD_640_ of 0.5, and 20 μl of this cell suspension was used to inoculate the wells of a 96-well polystyrene microtiter plate (Greiner Bio-One) containing 180 μl of LB medium. Wells containing sterile growth medium were used as negative controls. Plates were incubated at 37 °C from 4 to 48 h without agitation. For biofilm quantification, the culture media and unattached bacterial cells were removed from the wells by careful rinsing with water (three times, 200 μl for each rinse). Adherent bacteria were stained with 200 μl of a 1% crystal violet solution for 15 min at room temperature (50 ml of the solution was prepared by adding 1% [wt/vol] crystal violet in 10 ml of 95% ethanol to 40 ml of water containing 0.4 g of ammonium oxalate). After three gentle rinses with 200 μl of water each time, the dye associated with the attached cells was solubilized in 200 μl of 95% ethanol and the biofilm was quantified by measuring the absorbance of the solution at 600 nm (*A*_600nm_) in a microplate reader.

### Statistics

A non-parametric Mann-Whitney u-test was used to determine statistical significance of the observed variations (GraphPad Prism 7; GraphPad Software, CA). *P* ≤ 0.05 was considered statistically significant.

## Data Availability

All datasets generated for this study are included in the manuscript.
